# Downstream changes in firing regularity following damage to the early auditory system

**DOI:** 10.1186/1471-2202-16-S1-O11

**Published:** 2015-12-18

**Authors:** Dan FM Goodman, Alain de Cheveigné, Ian M Winter, Christian Lorenzi

**Affiliations:** 1 Department of Electrical and Electronic Engineering, Imperial College London, London, UK; 2 Laboratoire des Systèmes Perceptifs, Centre National de la Recherche Scientifique and École Normale Supérieure, Paris, France; 3 Centre for the Neural Basis of Hearing, The Physiological Laboratory, Department of Physiology, Development and Neuroscience, University of Cambridge, Cambridge, UK

## 

We demonstrate how an abstract mathematical model that approximates a wide range of more detailed models can be used to make predictions about hearing loss-related changes in neural behaviour.

One consequence of neurosensory hearing loss (noise-induced and aging-related) is a reduced ability to understand speech, particularly in noisy environments, and sometimes beyond what would be predicted from reduced audibility. Indeed, this type of speech deficit can occur in listeners with near-normal hearing thresholds [1]. A promising avenue of investigation to explain this comes from experimental results in mice showing that there can be a permanent loss of auditory nerve fibres (ANFs) following "temporary" noise-induced hearing loss (i.e. when thresholds return to normal after a few weeks) [2]. The downstream consequences of this loss of fibres has not yet been systematically investigated (although see [3]). We predict, using a theoretical analysis that applies to a wide range of neural models, that the regularity of the spike trains of many neurons in the cochlear nucleus (the next structure after the auditory nerve) will decrease following a reduction in the number of input cells.

We present a mathematical analysis of the stationary behaviour of "chopper" cells in the ventral cochlear nucleus, approximating them by a stochastic process that is entirely characterised by its mean, standard deviation and time constants. Furthermore, these constants can be straightforwardly related to physiologically significant parameters including the number of inputs and their average firing rates. From this approximation, we can compute the regularity of the chopper cell spike trains measured as the coefficient of variation of their interspike intervals (CV).

One simple prediction of this model is that when the intensity of a stimulus changes, leading to a change in the average firing rate of the ANF inputs, there will be a corresponding change in the regularity of the chopper cell spike train. This prediction poses problems for the widely used scheme for classifying chopper cells as sustained or transient based on their ongoing CVs as it implies that the classification could be level-dependent. We present a re-analysis of an existing experimental data set that demonstrates that ongoing CV is indeed level-dependent in the majority of chopper cells, and that in some cells (>7%) this leads to a level-dependence in their classification.

Assuming a homeostatic regulation of long term firing rates, a loss of ANFs will lead to an increase in the standard deviation of the stochastic process and a consequent increase in the CV of the chopper cell. Some choppers that were previously classified as sustained will become transient, a substantial change in their behaviour that is highly likely to disrupt auditory processing. While the function of chopper cells is still debated, one suggested role is in the coding of temporal envelope [4], which is widely agreed to be essential for understanding speech. Loss of ANFs could therefore lead to a disruption of the processing of temporal envelope, and consequently degrade speech intelligibility. We briefly conclude by discussing the challenges of testing this hypothesis experimentally.
